# National review of end-of-life care withdrawal guidelines for non-invasive advanced respiratory support using document analysis

**DOI:** 10.1136/bmjopen-2024-089617

**Published:** 2024-10-15

**Authors:** David Wenzel, Thomas Jeffery, Rachel Davies, Jennifer Creese, Eleanor Wilson, Christina Faull

**Affiliations:** 1 Population Health Sciences, University of Leicester, Leicester, UK; 2 Palliative Care, LOROS Hospice, Leicester, UK; 3 University Hospitals of Leicester, Leicester, UK; 4 UK Palliative Care Reseach Collaborative, Bristol, UK; 5 School of Health Sciences, University of Nottingham, Nottingham, UK; 6 LOROS, Leicester, UK

**Keywords:** palliative care, pulmonary disease, chronic airways disease, protocols & guidelines, adult palliative care

## Abstract

**Abstract:**

**Objectives:**

This study aims to understand the breadth of practice around end-of-life withdrawal of non-invasive advanced respiratory support (encompassing both continuous positive airway pressure and non-invasive ventilation) by analysing NHS-published guidelines and guidance for clinicians. This study seeks validity in the guidelines through a confluence of findings and reassurance of practice despite having little to no high-quality research to inform the content of the guidelines. Ultimately, where discordance is found between guidelines, there will be a strong mandate for future research.

**Methods:**

Guidelines were gathered through snowball sampling and analysed using document analysis techniques. Analysis was mixed in inductive and deductive and facilitated across several authors using framework analysis. 20 guidelines were analysed but saturation was found after 15. Further guidelines were analysed beyond saturation to provide reassurance of the endpoint of the study.

**Results:**

There were common components to the guidelines presented as themes: legal and ethical frameworks, decision-making around withdrawal, the process of withdrawal, post-withdrawal care and when to contact palliative care. There were significant areas of confluence, where multiple guidelines were in agreement on best practice. However, there was significant discordance in some key areas including the use of post-withdrawal oxygen therapy and pressure weaning practice.

**Conclusion:**

This study provides reassurance through a confluence of findings for the majority of withdrawal practices. However, key areas of discordance highlight an urgent need for further research to support clinicians, patients and their families during challenging clinical events.

**STRENGTHS AND LIMITATIONS OF THIS STUDY**

- The analysis of the data followed Bowen’s established approach for document analysis.
- Multiple authors contributed to analysis, enhancing the reliability of the findings.
- 20% of UK National Health Service Trusts were represented in the data.
- However, not all relevant guidelines may have been collected, which may result in limited generalisability.
- The prevalence and distribution of guidelines were not assessed in this study, nor was the quality of individual guidelines.

## Introduction

 The use of non-invasive advanced respiratory support (NARS), including non-invasive ventilation (NIV) and continuous positive airway pressure (CPAP), is widespread in modern healthcare settings. NARS is used in the treatment of chronic obstructive pulmonary disease, heart failure, obesity hypoventilation syndrome, obstructive sleep apnoea and neuromuscular disorders (including motor neuron disease (MND)).^1^ Many of these conditions have high mortality rates, but there is no clear clinical consensus for how treatment of these conditions should be withdrawn if ineffective. The COVID-19 pandemic saw mass use of NARS among those both with, and without, pre-existing conditions. The high mortality rate of COVID-19, where needing NARS was associated with a mortality rate between 30% and 90% among select patient groups,^2 3^ brought the need for clinical guidelines into national focus. In clinical practice, NARS therapy is felt to have failed if the patient’s clinical condition fails to improve or the patient requests withdrawal. While good quality palliative care can be administered with the NARS interface (most often a mask) in situ^4^ and may even aid symptom control,^5^ there is a significant treatment burden to contend with.^6^ Little is known about the experience of dying using NARS,^7^ and therefore, there is little high-level information on which to base guidelines for withdrawing NARS during end-of-life care. The pandemic highlighted these absences in the literature, and many National Health Service (NHS) Trusts published local end-of-life care NARS withdrawal guidelines to support teams caring for patients dying despite NARS. These guidelines often initially pertained to COVID-19, but some have been adapted and expanded to inform the care of patients dying from respiratory failure generally. These guidelines represent local expert opinion, but there is no requirement to standardise them nationally, which may result in regional variation in the way that care is provided. These guidelines are highly specific to the withdrawal of NARS which is often carried out outside of intensive care settings, in more frail patients and with less prewithdrawal sedation. The potential for NARS to act as symptom control also represents a very different situation to the withdrawal of other forms of ‘life support’ like endotracheal ventilation. This study explored the content of these guidelines in a proceduralised way using document analysis methodology.^8^ Bowen maintains that areas of concordance between concurrently written documents can be treated as adding validity to their content, reassuring us of our practice. Areas of discordance may highlight meaningful areas of research need. These documents are created to be reflective of our values around end-of-life care but also actively shape our values as well. These guidelines are societal artefacts that inform practice, permitting and promoting some actions while preventing others. It is important to understand their content, not only as NARS withdrawal may represent the final opportunity to give our patients excellent end-of-life care, but because the way someone dies can meaningfully alter the grieving journey of their loved ones.^9^

## Methods

### Study design

The study used Bowen’s (2009) document analysis, whereby eligible guidelines were qualitatively reviewed to seek areas of concordance or discordance. Guidelines, policies and Trust published ‘advice’ documentation were included for analysis if they supported a clinician’s care of a patient receiving NARS (CPAP and or NIV) at the end-of-life where treatment was felt to have become ineffective and needed to be withdrawn. If documents make comments on withdrawal but advocated for NARS to stay in place until death, these were included for analysis. The aim of the analysis is to understand these documents, and critically interrogate common or contradictory themes within and across them, rather than to highlight or denigrate outliers of practice.

### Data collection

Guidelines were gathered from snowball sampling; a call for guidelines was emailed to the committee members of the UK Palliative Care Research Collaborative (UKPRC). The UKPRC is a national research collaborative made up of palliative medicine registrars working at geographical locations across the UK. Recipients were encouraged to send in guidelines and pass on the study information to any relevant or interested parties. In addition, freedom of information requests were sent to Trusts in geographical areas of the country where no guidelines had thus far been collected. Collection of the guidelines took place between February 2024 and May 2024 concurrently with data analysis—at this time there were 215 acute hospital Trusts in England, 7 equivalent organisations in Wales, 14 in Scotland and 6 in Northern Ireland who may use such guidelines. All contributing Trusts are credited in online supplemental appendix A, but we intentionally do not link Trusts to findings in our analysis.

### Data extraction and analysis

Three authors (DW, TJ and RD) undertook the analysis, using document analysis, whereby selected guidelines are appraised, and data are synthesised directly from the documents which are then presented as themes. The coordination of authors was facilitated by the generation of an iterative framework which tracked relevant emergent themes.^10^ Analysis was a mix of inductive and deductive, aided by the clinical backgrounds of the authors (DW/RD in palliative care and TJ in internal medicine).

**Figure 1:**
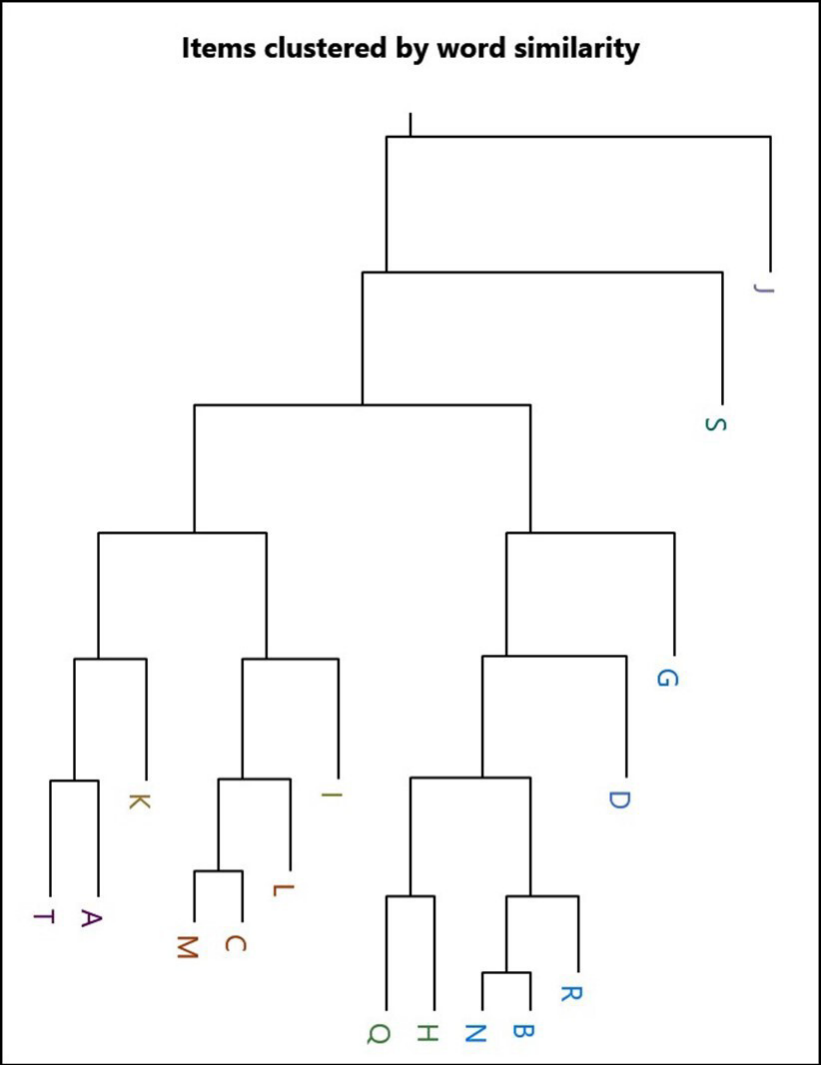
Dendrogram from Jaccard’s coefficient cluster analysis.

Guidelines were selected purposively for initial analysis to create the greatest variation in document content—this was achieved by reading 10 guidelines and selecting those with the greatest variation in length, medication doses, clinical reasoning and the actual process of withdrawal. This purposive selection was aided using a Jaccard’s analysis and dendrogram (figure 1) that demonstrates textual similarity between the documents. The first guideline was coanalysed by DW, TJ and RD to produce a framework. This framework was applied independently by DW to three further guidelines. Areas of development need were discussed and a further framework was developed. The second framework was applied independently by all authors to assess its efficacy. Once consensus was reached, DW applied the framework to further guidelines seeking thematic saturation or the review of 20 guidelines, whichever came later. Thematic saturation was agreed after 15 guidelines, but analysis continued to 20 to ensure saturation.

Final themes were generated by DW, TJ and RD. Document codes of a similar nature, such as ‘weaning pressure settings’ and ‘give medications’, were grouped into themes that represented the codes. Broadly, themes and codes could be narrated into a timeline of expected events.

### Patient and public involvement

This study is part of a larger corpus of work that has been informed and developed alongside extensive PPI involvement. Given the focus of the analytic work, PPI input for this study was limited to agreeing on the acceptability and shape of the research question. The PPI group was supportive of the study’s aims.

## Results

21 guidelines were gathered from geographically distinct areas of the country. Nine from FOI requests (of 18 requests sent), 12 from email cascade. Of note, no guidelines originated from Northern Ireland despite extensive liaison with Northern Irish clinicians and multiple freedom of information requests. Guidelines originated from England (17), Scotland (3) and Wales (1). Three guidelines were attributed to guideline collectives, aggregates of NHS Trusts which share common guidelines and policies. However, Trusts covered by guidelines from collectives sometimes still publish their own separate and distinct guidelines. Including deduplication of Trusts that published local guidelines while being covered by regional guidelines, 49 Trusts are represented in the results (17 local guidelines, 3 regional covering 37 Trusts with 5 duplicates equating to 20.2% of 242 national Trusts). The titles of the guidelines are summarised in online supplemental appendix B. Some guidelines pertained to respiratory failure, while others specifically addressed COVID-19. One guideline is reported as temporary but is still in use. None of the guidelines included guidance around endotracheal ventilation withdrawal and four specifically referenced they were for use only at a ward-based level of care.

## Findings

There was universal commonality among the guidelines, promoting compassionate, individualised care that incorporated significant family input into withdrawal decisions. The guidelines broadly had common features which are presented here as the themes: legal and ethical frameworks, decision-making around withdrawal, the process of withdrawal, post-withdrawal care and when to contact palliative care. Though not every guideline contained features from every theme, there were many areas of concordance where guidance was given. However, within these themed areas, there were key issues where discordance was identified. The dendrogram (figure 1) demonstrated objective similarity between some of the source documents. Guideline C is a regional document and M and L independent Trusts within that region have published their own guidelines, and their similarity is clearly demonstrated. Guideline B is similarly regional with guideline N appearing within its geographical area. However, the text of guideline R is as similar to B/N as L is to C/M despite the fact that R is in a geographically distant location. There was clear evidence of geographically remote Trusts informing their guidelines from a national collaboration of work.

### Legal and ethical frameworks

Most of the guidelines included consideration of the legal and ethical frameworks through which the withdrawal of NARS was undertaken. Of particular note, half of the guidelines stated that withdrawal of NARS does not represent euthanasia. Some explicitly made this statement:

‘Withdrawal of respiratory support is not assisted dying/suicide or euthanasia’ Guideline H

Others did so less directly:

The withdrawal of treatment, whether at the request of a competent patient or following consideration of best interests in a patient who lacks capacity, allows the underlying disease to take its natural course, which has been temporarily delayed by the NIV Guideline A

The lack of national guidance was noted in some guidelines, and many used existing research/academic literature and other, indirectly supportive, guidelines in the construction of their own:

There are currently no national guidelines on recommended sedation used to proactively manage symptoms for withdrawal of NIV and practices can vary across different trusts and between different consultants Guideline I

Other documents referenced are listed in online supplemental appendix C. Reference to other sources used to inform the principles of care were included in guidelines by B, D, G, H, I, K, N, O, R and T. Many of the guidelines appeared to have been informed by the Association of Palliative Medicine (APM) guidance for the withdrawal of NIV at the request of a patient with MND.^11^ In some instances, this was an explicit association, but for others, the association was inferred by the similarity of content. Some did acknowledge the difficulties in translating this guidance into an acute setting with different illnesses:

The focus of assisted ventilation withdrawal has been in patients with deteriorating neuromuscular conditions, such as MND… The disease trajectory of these conditions has allowed time for thought and discussion, reaching conclusions and a plan in line with the patient’s wishes. The process and best practice developed by treating these patient groups will now need to be transferred to a different setting and disease trajectory. Guideline N

Guidelines often used reference to case or statute law to support them, particularly in providing clarity that withdrawal of unwanted and/or medically futile treatment is not euthanasia. These references were to ‘Aintree V James 2013’ by guideline H, the ‘Mental Capacity Act 2005’ by A, B, N and R and the ‘Human Rights Act 1998’ by B, N and R.

### Decision-making around withdrawal

All but one of the guidelines included comments on best practices for how to make decisions around withdrawal. Three guidelines, all nested within larger NIV guidelines, primarily made recommendations on decision-making rather than guidance on the process of withdrawal. One guideline only included advice on the process of withdrawal with no comments on decision-making.

Fewer than half of the guidelines described leaving the mask in situ until death has occurred, but where this was advocated for it was often to palliate the sensation of breathlessness:

Respiratory support may in itself be viewed as a palliative treatment of dyspnoea Guideline R

Advice and guidance on decision-making were extensive throughout the guidelines with few areas of discordance between them. Some guidelines recommended specific language or approaches to end-of-life conversations, others did not. Almost all guidelines advocated for family involvement, patient involvement, assessments of capacity and prioritisation of individualised care.

It is recommended to include family/loved ones in the decision making process, particularly if it is anticipated that the patient will die soon Guideline H

At all times, care should be tailored to an individual patient’s needs, and effective communication between professional [sic] and patient+/-family is paramount Guideline G

Even in guidelines that did not advise on specific language to communicate withdrawal decisions to patients and their families, most guidelines included advice on what the most important factors were to communicate. These included the prognostic uncertainty of how long a patient may live after withdrawal, what to expect from the withdrawal itself and the medical aim of care now being the relief of distressing physical symptoms:

The plan of care will now focus on those interventions that prioritise the comfort of the patient… Warn family that although patients often deteriorate and die within minutes to hours, occasionally this can take longer. Guideline L

What to expect during the process of removal including who will remove the mask and who will give the medications. Guideline Q

### The process of withdrawal

There was variation in the guidance for the process of withdrawal, particularly around the level of sedation and medication recommendations. Some guidelines specified the patients should be heavily sedated prior to withdrawal, while others advised a more nuanced approach, focusing on symptom control without specific reference to sedation levels:

The patient should achieve a reduced conscious level with no response to voice or painful stimuli. Repeat the 2 mg bolus doses until this has been achieved. Guideline N

The patient should be settled and comfortable Guideline A

The recommended doses of medications, therefore, varied, as well as the interval between repeat doses, summarised in online supplemental appendix D. There was more uniformity in the choice of medication, with all but one guideline using morphine and midazolam to control symptoms as a first line. The remaining guideline advocated for the use of levomepromazine as an initial medication, specifically citing its lack of effect on respiratory drive. All guidelines started the process of withdrawal with the administration of an anticipatory dose of their chosen medications and, where advice was found, it was unanimous that symptoms should be controlled prior to withdrawal.

If the patient remains settled and sedated, then: remove the mask Guideline B

Some guidelines highlighted that the NARS itself may be the cause of the distress, in which case faster withdrawal may be appropriate:

If patient is not tolerating the respiratory support and this is adding to symptom burden this may not be possible and in this case [the] key is to remove as quickly as possible at the same time as giving medication for symptom relief. Guideline Q

Once patients’ symptoms were controlled, there was significant discordance in the approach to removing the NARS. Some guidelines simply referred to removing the mask, without specifically outlining how. The remaining guidelines fell into two groups, those that advocated for weaning pressures/O2 flows and those that specifically counselled against it:

For NIV, reduce pressure support by 50% increments until symptom controlled removal is possible Guideline R

It is not common practice to wean patients from NIV/CPAP/HFO2 by reducing ventilator settings or O2 percentage as this can prolong the dying process and increase the discomfort experienced by the patient. Guideline L

All of the guidelines that advised on the withdrawal process advocated for pausing and re-evaluating symptom control regularly, giving further medications if indicated.

Observe for effect [of initial medication] allow 30–60 mins. If symptoms controlled and therapeutic sedation achieved wean NIV/CPAP. If symptoms of distress evidence on weaning—pause and repeat stat doses above. Guideline S

Once the mask is withdrawn, most guidelines do not describe circumstances in which the NARS interface may be put back on, however, if symptoms cannot be controlled this was advocated by three guidelines. Two highlighted this was not a preferable action given the distress it may cause to families.

Try and avoid repeatedly replacing interfaces once withdrawal commenced [sic] as relatives may find this distressing Guideline Q

if a patient becomes distressed after NIV is removed despite sedation then you should replace NIV and discuss repeating and re-evaluating appropriate sedation. However, this can be very distressing for the patient and their families Guideline I

Other components of withdrawal appeared inconsistently across guidelines but without discordance. These were descriptions of how many/which staff should be involved; advising, if possible, to wait until normal working hours; removing monitoring equipment; silencing alarms and assessing dependency prior to withdrawal. The assessment of NARS dependency (how quickly a patient became symptomatic with the mask off for personal care) was used at times to modify the withdrawal process. If patients are able to tolerate time off the mask, no medications may be needed, but highly dependent patients may require more medication.

Best outcomes result from consideration of patients in two groups related to level of ventilator dependence. [Highly dependent patients] extremely likely to become very breathless and or distressed within minutes of removal Guideline K

Guideline K advocated for the use of a continuous subcutaneous infusion of medication before withdrawal to be used for patients who were highly dependent, which could be omitted for less-dependent patients. None of the reviewed guidelines referenced assessment tools or clinical scales to aid in the assessment of agitation or symptom burden.

### Post-withdrawal care

There was broad agreement across the guidelines that further doses of medication may be required following withdrawal if symptoms recur. Where use of these medications was advocated, it was at the same dose as medications used during the withdrawal process (summarised in online supplemental appendix D). Several guidelines advocated for the use of continuous subcutaneous infusion of medications if the patient lived for a long time following the withdrawal of NARS:

If a patient survives off their ventilatory support for an hour, a syringe driver should be commenced to ensure ongoing symptom control. Doses will be based on the amount needed for symptom control during the initial withdrawal Guideline R

There was one significant area of discordance in the post-withdrawal care of patients, related to the use of post-withdrawal oxygen. Some guidelines viewed it to be standard practice:

Administer Oxygen (Hudson Face mask/Nasal Cannula) [sic] adjust the rate aiming for comfort… aim to reduce oxygen to lowest tolerated rate Guideline D

Others made mention of post-withdrawal oxygen without guidance as to when it may be appropriate:

Will the NIV mask be replaced by an oxygen mask? This should be discussed and a decision made for each patient on an individual basis Guideline A

Two guidelines, B and N, explained the aim of post-withdrawal oxygen was to prevent cyanosis.

Hypoxia can develop rapidly, this may add to feelings of dyspnoea and distress. It can also be distressing for the family if their loved one becomes rapidly cyanosed Guideline N

One guideline advocates that oxygen should not be used in the post-withdrawal period:

Switching to O2 via venturi mask can prolong the dying process and potentially increase distress for all involved. If the idea of having no oxygen causes distress for family, you could consider a small flow of supplemental oxygen via nasal prongs Guideline L

Other components of post-withdrawal care that appeared in some guidelines but not others included holistic/spiritual care and updating families. Around half of the guidelines highlighted specifically the need to debrief staff following a withdrawal:

Check in with colleagues who were involved with the withdrawal afterwards. Take a break if needed. Reflect: anything that went well/lessons to be learnt? Guideline H

### When to contact palliative care

Not all guidelines had descriptions of their authors’ backgrounds and specialties. An authoring specialty was identified for 15 of 20, of which palliative care specialists contributed to 12 guidelines, having solely authored 7. One guideline was authored by a critical care specialist and three by respiratory medicine. Only one guideline stated that the input of a palliative care specialist was required for all patients as routine care:

for all patients a palliative care consultant and [clinical nurse specialist]… should be involved at an early stage [of withdrawal] Guideline A

Among the remaining guidelines, 12 spoke about involving the palliative care team—most often related to specific complex circumstances. Most commonly, this is related to medications, either selecting an opioid in non-typical circumstances (eg, hepatic or renal failure) or titrating medications against previous exposure. Guidelines also suggested contacting palliative care if patients did not respond to medications:

If no effect seen from repeated PRN doses, pause process… Call specialist palliative care team for advice Guideline H

If the patient requires more than 20mg of morphine and midazolam and remains unsettled then… contact palliative care for advice Guideline B

Other reasons to contact palliative care included support with communication and the psychological care of staff involved in withdrawal care:

discussions ideally should include for palliative care [clinical nurse specialist] Guideline A

we know that withdrawal of respiratory support is an emotive topic. Physicians have reported anxiety and distress about the perception of withdrawing respiratory support… if help, advice and/or support are required with a complex case then please contact the palliative care team Guideline R

## Discussion

The guidelines demonstrated significant similarity and at times it was clear that the construction of one guideline had been informed by another. There was often no declaration within the guidelines that they had been adapted from another source. However, several did reference the APM’s ‘withdrawal of ventilation in MND’ guidelines as one of their sources.^11^

Given Jaccard’s analysis (figure 1) demonstrated such clear similarity in document content, it could be assumed that consensus for a national guideline may be easily generated. But although the guidelines, especially among geographically close sources, are broadly similar there are significant areas of discordance.

Guideline C, as a regional guideline, makes no mention of post-withdrawal oxygen therapy. However, guideline L strongly advocates against its use. The inclusion of this information despite the overall similarity of the local versus regional guidance suggests a strong local opinion. Neither guideline can claim stronger footing in the evidence base given the lack of good quality data in this area.^7^ This discordance creates a clear mandate for research. It is unusual in modern medicine for standard practice in one hospital to be identified as poor practice by another local trust. This speaks to the difficulty of providing safe and effective care for this vulnerable group of patients. This conflicting guidance can lead to confusion among practitioners, and this was a key factor behind the development of the national MND withdrawal guidance.^12^ A lack of support and guidance can also contribute to moral distress in this area of practice which is compounded by staff often feeling implicated in the death of a patient through their involvement in withdrawal.^13^

Though some guidelines acknowledged the emotional impact this care can have on staff, there was little developed guidance in these documents. Some guidelines discussed the need to debrief with staff, but only guideline R specified who may run this debriefing (the palliative care team). How the debrief should run, or when it should be undertaken, was omitted. It may be that further guidance is found in other documents, but its absence may also suggest an uncertainty around what structure of debriefing would bring the greatest benefit. The lack of structure in the staff care components of the documents, as well the omission from half of them, suggests a somewhat perfunctory acknowledgement of staff well-being.

Similarly, absent from all the guidelines is advice, support or recognition of the patients’ and their families’ psychological needs and the management of psychological trauma. Many guidelines refer to ‘distress’ and ‘anxiety’, but exploration of emotional need is limited beyond that. Further research is required to evaluate whether this is an unwritten part of withdrawal care (ie, not in the guidelines but present in practice) or an underserved aspect of our holistic duty. This is especially pertinent given the existing literature on how the manner of a person’s death may influence their grieving process or increase the risk of post-bereavement psychiatric illness.^14 15^

Managing symptoms in relation to withdrawal is a complex and under-researched topic which is typified by the lack of concordance around post-withdrawal oxygen and pressure weaning. It is unclear if, where NARS has failed to improve a patient’s condition, post-withdrawal oxygen can make a meaningful difference to oxygenation and cyanosis; it may instead be merely providing enough oxygenation to prolong the dying process. Guideline L highlights that the family may find not having post-withdrawal oxygen in place distressing, and suggests a small, almost homeopathic, dose of oxygen. Further exploration of this topic may reveal whether the common recommendation of post-withdrawal oxygen in guidelines is actually a way of managing staff distress around the same issues (ie, it is emotionally challenging for staff to feel they are doing ‘nothing’).

Outside of the physiological questions around whether post-withdrawal oxygen could meaningfully alter the length of dying or symptom control, it is important to consider the purpose of withdrawing NARS. If NARS is withdrawn not purely for symptom control, but to demedicalise the dying process, reintroducing post-withdrawal oxygen may obstruct this objective. The guidelines do reference both topics, but research to explore the efficacy of post-withdrawal oxygen, and qualitative explorations of the intention behind withdrawing, are needed before robust conclusions can be drawn. Ultimately, much of this research will need to be informed by work with bereaved loved ones who can give insight into patients’ final moments.

There was similar discordance around pressure weaning; those who weaned used it as an opportunity to test the depth of efficacy of symptom control before committing to withdrawing the mask completely. This contrasted with those who felt weaning prolonged the withdrawal process and dying experience. It could be viewed that weaning to test symptom control could prevent a sudden and unexpected increase in symptoms after withdrawal. If the patient becomes extremely distressed, this may increase the risk of needing to replace the mask—slowing the withdrawal and distressing patients and loved ones. There is likely no objective ‘best practice’ in this area of withdrawal, but it remains unusual to have one hospital’s best practice be actively counselled against by another.

The lack of robust evidence prevents guidelines from claiming authority from data, but the frequent description of other guidelines, case and statute law speaks to an attempt to derive authority from other sources. This appears to be an attempt to reassure staff about the use of these guidelines, and ‘permission’ to withdraw NARS. The frequent presence of euthanasia references across the guidelines may also speak to the guideline authors’ experiences of colleagues who erroneously believe this to be the case. This concept has been explored in existing literature and warrants ongoing consideration in staff training,^16^ including understanding of General Medical Council (GMC) guidance on withdrawal of treatment. Much of the legal and ethical framework applied to NARS withdrawal is also relevant to the withdrawal of endotracheal intubation and life support, which speaks to the gravity to which NARS withdrawal is held.

Despite NARS withdrawal being a complex end-of-life care experience, there is a clear thread throughout the guidelines which suggests that authors do not envisage this to be exclusively a palliative care service procedure. Instead, the role of palliative care is more in the complex cases or second-line medications, that is, this is a general medical issue, not specialist. This creates further consideration of educational requirements for the general physician.

There was discordance in the medications used, the doses administered and the levels of sedation that the medications were intended to achieve. Variations in medication requirements can occur even when a singular guideline is used, as was demonstrated following evaluation of medication doses after elective withdrawal in MND.^17^ The aim of all guidelines was the same despite discordance in medication utilisation: the withdrawal of NARS should represent a dignified and symptom-free moment of care.

Overall, there is a significant overlap between guidelines in the perception of good end-of-life care and this starts, in most instances, with a good withdrawal of NARS. A good withdrawal is inferred from the guidelines to start with extensive communication with family and patients and include senior doctors and the multidisciplinary team. This involvement should be present in both the decision-making and process of withdrawal. This is followed by a withdrawal process that focuses on symptom control and the relief of suffering during this final phase of treatment.

### Limitations

The study’s limitations include the potential incompleteness of the dataset, as not all relevant guidelines may have been collected, which may result in bias limiting generalisability of results. Additionally, the prevalence and distribution of the guidelines across different regions is unable to be determined as this was not assessed. Lastly, a systematic review formally assessing the quality of individual guidelines and studies was not performed.

## Conclusion

This study highlights significant areas of both concordance and discordance within UK guidelines for the withdrawal of NIV in end-of-life care. While there is widespread agreement on the importance of compassionate, individualised care, notable disparities—especially regarding post-withdrawal oxygen therapy—reveal a concerning lack of consensus, with some guidelines offering directly opposing recommendations. Furthermore, the study identifies significant gaps in the support provided to both patients’ families and healthcare staff, who may experience distress during the withdrawal process. The underemphasis on the role of emotional support and facilitating team debriefs exacerbates these challenges. These findings underscore the urgent need for a national set of standardised guidelines to ensure consistent, high-quality care across all settings, ultimately enhancing the experiences of both patients and healthcare professionals during this challenging phase of treatment.

## supplementary material

10.1136/bmjopen-2024-089617 online supplemental file 1

10.1136/bmjopen-2024-089617 online supplemental file 2

10.1136/bmjopen-2024-089617 online supplemental file 3

10.1136/bmjopen-2024-089617 online supplemental file 4

## Data Availability

Data are available on reasonable request.
