# Supplementary material for: National review of end-of-life care withdrawal guidelines for non-invasive advanced respiratory support using document analysis
Source: BMJ Open. 2024 Oct 15;14(10):e089617. doi: 10.1136/bmjopen-2024-089617 (PMC11481104; doi:10.1136/bmjopen-2024-089617)
Supplement: online supplemental file 1 [file bmjopen-14-10-s001.pdf]

Appendix A

ACKNOWLEDGEMENTS

Contributing NHS Trusts

South Tees Hospital NHS Foundation Trust

NHS Tayside

Specialist Palliative Care Audit and Guidelines Group – West Midlands

Right Decision Scotland

Kettering General Hospital NHS Foundation Trust

Leeds Teaching Hospitals NHS Trust

University Hospitals Bristol and Weston NHS Foundation Trust

Worcester Acute Hospitals NHS Trust

George Eliot Hospital

North Staffordshire Combined Healthcare NHS Trust

Sandwell and West Birmingham Hospitals NHS Trust

Buckinghamshire Healthcare NHS Trust

Hampshire Hospitals NHS Foundation Trust

NHS Lanarkshire

Berkshire Healthcare NHS Foundation Trust

North West Coast Strategic Clinical Networks

Royal Cornwall Hospitals NHS Trust

Norfolk and Suffolk NHS Foundation Trust

Royal Liverpool and Broadgreen University Hospitals NHS Trust

Swansea Bay University Health Board

Special Thanks

The UK Palliative Care Research Collaborative
