# Supplementary material for: National review of end-of-life care withdrawal guidelines for non-invasive advanced respiratory support using document analysis
Source: BMJ Open. 2024 Oct 15;14(10):e089617. doi: 10.1136/bmjopen-2024-089617 (PMC11481104; doi:10.1136/bmjopen-2024-089617)
Supplement: online supplemental file 2 [file bmjopen-14-10-s002.pdf]

Appendix B

Guideline	Guideline Code
Guidelines for withdrawing non-invasive ventilation (NIV) at end of life.	A
Withdrawal of assisted ventilation for hospital inpatients outside of critical care settings/ICU	B
Guidance for end-of-life care in a patient receiving NIV, CPAP or high flow oxygen in cases of Covid-19 respiratory failure	C
Trust guideline-non-invasive ventilation (NIV) for acute hypercapnic respiratory failure	D
Guidelines for the use of non-invasive ventilation (ward-based)	E
Clinical guidelines for non-invasive ventilation in acute respiratory failure.	F
Care of the dying patient (including patients with covid-19) guiding principles for good end of life care	G
Discontinuing (withdrawing) unwanted or ineffective high level respiratory support including CPAP and NIV	H
Guidelines for withdrawal of non-invasive ventilation (NIV)	I
Guidelines for medications when removing CPAP / NIV at the end of life	J
Guidance for withdrawal of respiratory support in conscious adults at the end of life within *** NHS trust	K
Withdrawal of respiratory support	L
NHS *** removal of respiratory support in covid HDU & guidance for EOLDC in a patient receiving NIV/CPAP (two guidelines)	M
Withdrawal of assisted ventilation for hospital inpatients	N
Symptom control, palliative care & referral guidelines for patients with chronic respiratory disease	O
Guidelines for the treatment of an emergency admission with acute hypercapnic respiratory failure including the use of non-invasive ventilation (NIV)	P
Recommendations for the withdrawal of respiratory support in the end-of-life care setting-NIV, CPAP and NHFO	Q
Withdrawal of non-invasive respiratory support for hospital inpatients in non-critical care settings clinical guideline	R
Use of ward-based CPAP in the management of covid-19 pneumonia and type I respiratory failure	S
Temporary non-invasive ventilation (NIV) withdrawal guidelines for use during the covid-19 outbreak	T
