# Supplementary material for: National review of end-of-life care withdrawal guidelines for non-invasive advanced respiratory support using document analysis
Source: BMJ Open. 2024 Oct 15;14(10):e089617. doi: 10.1136/bmjopen-2024-089617 (PMC11481104; doi:10.1136/bmjopen-2024-089617)
Supplement: online supplemental file 3 [file bmjopen-14-10-s003.pdf]

Appendix C

- “One chance to get it right – 5 priorities for care of the dying patient”.
- “End of life care strategy (2017) NICE quality standard for end of life care”.
- “Ambitions for palliative and end of life care (2021)”.
- “Withdrawal of assisted ventilation at the request of a patient with MND [association of palliative medicine]”.
- “GMC guidance: Treatment and care towards the end of life: good practice in decision making (2010)”.
- “More Care, Less Pathway (2013)”.
- “COVID-19 – Ethical Issues. A guidance Note (British Medical Association, 2020)”.
