# Supplementary material for: National review of end-of-life care withdrawal guidelines for non-invasive advanced respiratory support using document analysis
Source: BMJ Open. 2024 Oct 15;14(10):e089617. doi: 10.1136/bmjopen-2024-089617 (PMC11481104; doi:10.1136/bmjopen-2024-089617)
Supplement: online supplemental file 4 [file bmjopen-14-10-s004.pdf]

Guideline Code	Level of Sedation	Preferred Medication Route	Initial Medication and Dose	Time Interval Before Repeated PRN
**A**	So that symptoms are controlled	IV or SC	Morphine 15mg & Midazolam 10mg	10-15 minutes
**B**	Sedated	IV	Morphine 2mg & Midazolam 2mg	As needed
**C**	So that symptoms are controlled	IV or SC	SC and IV dose:<br>Morphine 2-5mg & Midazolam 2-5mg	IV: 3-5 minutes<br>SC: 10-15 minutes
**D**	Sedated	SC	Morphine 2.5-10mg & Midazolam 2.5-10mg	10-15 minutes
**G**	Not specified	SC	Morphine 5mg & Midazolam 5mg	20 minutes
**H**	So that symptoms are controlled/Light sedation	SC	Morphine 5mg & Midazolam 5mg	5 minutes
**I**	Not Specific	SC for initial medication, thereafter IV or SC	Levomepromazine 50-100mg with additional Morphine and Midazolam if needed	IV: 10 minutes<br>SC: 20-30 minutes
**J**	Not specified	SC	Morphine 5-10mg & Midazolam 5-10mg	20 minutes
**K**	Patient choice – symptoms controlled or sedated	IV	SC: Morphine 5 – 10mg & Midazolam 5-10mg<br>IV: Morphine 2.5 – 5mg & Midazolam 2.5-5mg	20-30 minutes
**L**	So that symptoms are controlled/light sedation	IV or SC	Morphine 2-5mg & Midazolam 2-5mg	IV: 3-5 minutes<br>SC: 10-15 minutes
**M**	So that symptoms are controlled/light sedation	IV or SC	Morphine 2-5mg & Midazolam 2-5mg	IV: 3-5 minutes<br>SC: 10-15 minutes
**N**	Sedated	IV	Morphine 2mg & Midazolam 2mg	As needed
**Q**	Not Specified	IV or SC	SC: Morphine 5 – 10mg & Midazolam 5-10mg<br>IV: Morphine 2.5 – 5mg & Midazolam 2.5-5mg	Routine: 20-30 minutes<br>Extreme Symptoms: 5 minutes

**R**	Sedated	IV	Morphine 2mg & Midazolam 2mg	As needed
**S**	Sedated	IV	Morphine 10mg & Midazolam 10mg	30-60 minutes
**T**	Patient choice – symptoms controlled or sedated	IV or SC	Morphine 5 – 10mg & Midazolam 5-10mg	10-15 minutes
